# Structural effects of intra-articular TGF-β1 in moderate to advanced knee osteoarthritis: MRI-based assessment in a randomized controlled trial

**DOI:** 10.1186/s12891-017-1830-8

**Published:** 2017-11-16

**Authors:** A. Guermazi, G. Kalsi, J. Niu, M. D. Crema, R. O. Copeland, A. Orlando, M. J. Noh, F. W. Roemer

**Affiliations:** 1 Department of Radiology, Quantitative Imaging Center, Boston University School of Medicine, 820 Harrison Avenue, FGH Building, 3rd Floor, Boston, MA USA; 2 TissueGene, Rockville, MD USA; 3 Baylor College of Medicine, Houston, TX USA

**Keywords:** Tgf-β1, Osteoarthritis, MRI, Randomized controlled trial

## Abstract

**Background:**

To determine effects of allogeneic human chondrocytes expressing TGF-β1 (TG-C) on structural progression of MRI features of knee osteoarthritis over a 1 year period.

**Methods:**

This phase II randomized controlled trial of TG-C included patients with moderate to advanced osteoarthritis. Patients were randomized to receive an intraarticular 3:1 mixture of non-transduced allogeneic human chondrocytes and TG-C or placebo. 3 T MRI was acquired for all patients at baseline and follow-up (3, 6 and 12 months). MRIs were assessed using the WORMS system including cartilage damage, bone marrow lesions (BMLs), meniscal damage/extrusion, Hoffa-, effusion-synovitis, and osteophytes. Analyses were performed on a whole knee level, compartmental level, and subregional level. Binary logistic regression with Generalized Estimating Equation was used to compare risks of progression, adjusting for baseline age and gender. Mann − Whitney − Wilcoxon tests were used to assess differences for continuous variables.

**Results:**

Fifty-seven Patients were included in the TG-C group and 29 in the placebo group. At 12 months, knees in the TG-C group showed less progression of cartilage damage compared to placebo on a whole knee level (34.6% vs. 47.9%; adjusted RR 0.7, 95%CI [0.5–1.1], *p* = 0.077). Less progression of Hoffa-synovitis and effusion-synovitis was observed in the TG-C group compared to placebo (9.6% vs. 21.1%, adjusted RR 0.5, 95%CI [0.2,1.2], *p* = 0.115). No statistically significant differences were seen for BMLs, meniscal damage and osteophytes.

**Conclusions:**

Intraarticular treatment with TG-C showed fewer patients in the treated group with progression in structural OA features and other MRI-defined inflammatory markers such as Hoffa-synovitis and effusion-synovitis. However, no differences were observed in regard to progression of BMLs and meniscal damage, or hypertrophic osteophyte formation.

**Trial registration:**

NCT01221441.Registered 13th October, 2010

## Background

TGF-β proteins induce osteogenesis and chondrogenesis, and play a role in cell growth, differentiation, and extracellular matrix protein synthesis [1]. TGF-β stimulates proteoglycan synthesis and chondrocyte proliferation, and may also have anti-inflammatory and immunosuppressive characteristics [1]. A new treatment approach for knee osteoarthritis (OA) involves intraarticular administration of human chondrocytes transduced with a viral vector containing the gene for TGF-β1 transcription. Recently, a preliminary evaluation of the efficacy of non-transduced allogeneic human chondrocytes and allogeneic human chondrocytes virally transduced to express TGF-β1 (TG-C) was done. TG-C is made of human chondrocytes which has immunosuppressive effect and is grown from tissue obtained from a polydactyly finger of a single infant donor [2]. TG-C represents a cell-mediated cytokine gene therapy approach for local intra-articular administration in patients with OA. TG-C showed positive effects on pain levels in patients with moderate to advanced knee OA, as demonstrated by the visual analogue scale (VAS) and International Knee Documentation Committee (IKDC) scores at 1 year follow-up compared to the control cohort [3]. Patients receiving TG-C had less knee pain, and they were less likely to need analgesics compared to placebo. However, effects of TG-C on MRI-assessed structural changes in knee joint tissues have only been assessed in a single study demonstrating mixed results after 12 months, i.e. those who received low-dose TG-C showed worsening mean MRI score of cartilage signal intensity, while those who had high-dose TG-C showed worsening mean MRI scores in bone surface osteophytes and periarticular inflammation [4].

Our study aimed to assess effects of intraarticular TG-C on structural progression of knee OA features based on semi-quantitative MRI evaluation compared with placebo during 12-months of follow-up period.

## Method

### Subject inclusion and exclusion

Our study was a multi-center double-blind placebo-controlled phase II randomized clinical trial (ClinicalTrials.gov identifier: NCT01221441.Registered 13th October, 2010). It was conducted in accordance with the International Conference on Harmonization Tripartite Guideline, Guideline for Good Clinical Practice, ethical principles with origin in the Declaration of Helsinki, as well as the USA Code of Federal Regulations. Patient recruitment started in May 2011 and ended in October 2013. Institutional review board ethical approval was obtained from all five recruitment sites – Center for Joint Preservation and Reconstruction, Baltimore, MD); Commonwealth Orthopedics, Arlington, PA; Advent Orthopaedics and Rehabilitation LLC, Pinellas Park, FL; University Orthopedics Center, State College, PA; and The Rothman Institute, Philadelphia, PA. We included both male and female subjects aged between 18 and 70 years; body mass index (BMI) between 18.5 and 45.5 kg/m^2^; grade 3 radiographic knee OA as determined by the criteria of Kellgren and Lawrence [5] and pain symptoms for more than four consecutive months with an intensity of ≥ 40 and ≤ 90 on the 100-mm VAS. Subjects were generally healthy based on physical examination, normal blood work including hematology and serum chemistry, and urinalysis. All laboratory values were within 20% of normal ranges. All patients had negative history of significant organ system disorders. Written informed consent was obtained from all subjects after the nature of the study was fully explained and understood by them.

Exclusion criteria included: Patients taking non-steroidal anti-inflammatory medications within 14 days of baseline visit unless washed out; Patients taking steroidal anti-inflammatory medications within 2 months of baseline visit; Patients with a recent (within 1 year) history of drug abuse and/or a positive urine drug test at the time of screening; Patients receiving injections to the treated knee within 2 months prior to study entry; Patients who had contraindications for 3 T MRI; Patients who were pregnant or currently breast-feeding; Patients with a history of systemic, rheumatic or inflammatory disease or chondrocalcinosis, hemochromatosis, inflammatory arthritis, necrosis of the femoral condyle, arthropathy of the knee associated with juxta-articular Paget’s disease of the femur or tibia, ochronosis, hemophilic arthropathy, infectious arthritis, Charcot’s knee joint, villonodular synovitis, synovial chondromatosis, and/or history of inflammatory arthropathy; Patients with ongoing infectious disease, including HIV and hepatitis B or C; Patients with clinically significant cardiovascular, renal, hepatic, endocrine disease, cancer, or Type I diabetes; Patients participating in a study of an experimental drug or medical device within 30 days of study entry; Patients that were unable to comply with the requisite study follow-up and not able to complete all of the follow-up office visits and 3 T MRI exams.

### Treatment

Patients were randomized to receive a 3:1 mixture of non-transduced allogeneic human chondrocytes and allogeneic human chondrocytes virally transduced to express TGF-β1 (TG-C) (TissueGene-C; TissueGene Inc., Rockville, Maryland, USA) or placebo (2 ml normal saline 0.9%). Details of the randomization procedure and determination of the sample size have been published previously [3]. TG-C was derived from a single human donor, grown from cartilage tissue from an infant polydactyly finger. Absence of viruses and other adventitious donor agents and the cell line were tested. Prior to TG-C or placebo administration, synovial fluid was aspirated from the patients’ knee joints. TG-C or placebo was then injected intra-articularly using an 18 gauge needle with an inferolateral or inferomedial approach while the knee was flexed to 90-degrees. Injection was performed over about 10 s to avoid shearing of cells. Both patients and physicians were blinded to drug/placebo status.

### Magnetic resonance imaging acquisition and interpretation

All patients underwent 3 T MRI at baseline and follow-up visits (3, 6 and 12 months) using a dedicated knee coil and the following protocol (triplanar intermediate-weighted fat suppressed sequences: TE 30–40 msec, TR 3600–4000 msec, 14 cm field of view, slice thickness 3 mm, 1 excitation, no phase wrap). MRIs were read by one expert musculoskeletal radiologist (AG, 18 years of experience in semiquantitative MRI assessment of OA) in sequential order - unblinded to the time sequence of MRI but blinded to all clinical information including treatment - using the modified semi-quantitative Whole Organ MRI Scoring (WORMS) system [6] including assessment of cartilage morphology (grade 0 = normal thickness and signal; grade 1 = normal thickness but increased signal on T2-weighted images; grade 2.0 = partial-thickness focal defect < 1 cm in greatest width; grade 2.5 = full-thickness focal defect < 1 cm in greatest width; grade 3 = multiple areas of partial-thickness (Grade 2.0) defects intermixed with areas of normal thickness, or a Grade 2.0 defect wider than 1 cm but < 75% of the region; grade 4 = diffuse (≥ 75% of the region) partial-thickness loss; grade 5 = multiple areas of full thickness loss (grade 2.5) or a grade 2.5 lesion wider than 1 cm but < 75% of the region; grade 6 = diffuse (≥ 75% of the region) full-thickness loss.), bone marrow lesions (BMLs – grade 0 = none;grade 1 = < 25% of the region; grade 2 = 25% to 50% of the region; grade 3 = > 50% of the region.), meniscal damage (grade 0 = intact; grade 1 = minor radial tear or parrot-beak tear; grade 2 = nondisplaced tear or prior surgical repair; grade 3 = displaced tear or partial resection; grade 4 = complete maceration/destruction or complete resection) and meniscal extrusion (grade 0 = < 2 mm; Grade 1 = 2–2.9 mm; grade 2 = 3–4.9 mm; grade 3 = > 5 mm), and inflammatory markers of disease (Hoffa-synovitis, grade 0 = normal; grade 1 = mild; grade 2 = moderate; grade 3 = severe, and effusion-synovitis, grade 0 = normal; grade 1 = < 33% of maximum potential distention; grade 2 = 33%–66% of maximum potential distention; grade 3 = > 66% of maximum potential distention.). Longitudinal changes were recorded including within-grade changes.

As TGF-β1 is considerd to be an anabolic agent, osteophyte presence and severity (grade 0–7) and change over time were additionally evaluated to compare rates of progression of osteophyte formation between groups.

### Analytic approach

Data analyses were carried out in three ways using the data at baseline and at 12-month follow-up only: First, using ‘delta-subregional’ approach, the number of subregions showing progression (score increase at 12-month follow-up), no change, or improvement (score decrease at 12-month follow-up), respectively, were added to give a single score for each knee. There were a total of 14 subregions in the knee by WORMS definition (5 subregions each for the medial and lateral tibiofemoral [MTF, LTF] and 4 subregions for the patello-femoral [PF] compartment). For example, if 4 subregions show progression (delta [+1] × 4 = [+4]), 8 subregions show improvement (delta [−1] × 8 = [−8]) and 2 subregions show no change (delta 0), the ‘delta-subregion change’ for this knee would be ‘-4’ (4 + [−8] = [−4]). Second, using ‘delta-sum’ approach, the absolute scores of all subregions were added within each compartment or within the whole knee. For these two approaches, progression was defined as overall delta > 0. Third, progression was defined as an increase in score in any of the subregions.

Binary logistic regression with Generalizing Estimating Equations was performed to assess relative risks of progression comparing data at baseline and 12-month follow-up, adjusting for baseline age and gender. Moreover, Mann − Whitney − Wilcoxon tests were used to evaluate differences of the continuous variables between treatment and placebo groups. All statistical analyses were carried out using SAS for Windows, version 9.1. Statistical significance was defined at *p* < 0.05.

## Results

TG-C group had 57 patients and placebo group had 29 patients. Baseline demographic characteristics of the TG-C and placebo groups were comparable without statistically significant differences regading age (55.9 ± 7.9 vs. 56.6 ± 9.4 years) and gender (37 [64.9%] vs. 17 [58.6%] female). There was no statistically significant difference between two groups regarding baseline summary score.

Regarding change in cartilage morphology from baseline to 12 months, a lower proportion of knees showed progression of cartilage damage (progression in any subregion) in the treatment group on a whole knee level with a trend towards statistical significance (34.6% vs. 47.9%; adjusted RR 0.7, 95% CI [0.5–1.1], *p* = 0.077). The delta-sum approach or delta-subregional approach showed no statistically significant differences between two groups on a whole knee or compartmental level (Table 1).

**Table 1:** Presence and relative risk of progression of cartilage damage, Hoffa-synovitis and effusion-synovitis in different knee compartments for all visits combined from baseline to 12 months

		Number of subjects who had follow-up visits and included in analysis		Number of subjects with MRI progression		Analysis adjusted for age and gender	
*Compartment*	*Definition*	*T* ^*a*^	*P*	*T*	*P*	*RR (95% CI)*	*p-value*
Knee	Progression in any subregion	133	71	46 (34.6%)	34 (47.9%)	0.7 (0.5,1.1)	0.077
	Delta Subregion > 0	123	71	43 (35.0%)	32 (45.1%)	0.8 (0.5,1.2)	0.207
	Delta Sum > 0	123	71	26 (21.1%)	23 (32.4%)	0.6 (0.3,1.2)	0.176
Lateral TFJ	Progression in any subregion	133	71	20 (15.0%)	12 (16.9%)	0.8 (0.3,1.9)	0.617
	Delta Subregion > 0	128	71	20 (15.6%)	12 (16.9%)	0.8 (0.4,1.9)	0.680
	Delta Sum > 0	128	71	13 (10.2%)	10 (14.1%)	0.7 (0.2,1.9)	0.434
Medial TFJ	Progression in any subregion	133	71	23 (17.3%)	12 (16.9%)	1.1 (0.5,2.5)	0.767
	Delta Subregion > 0	133	71	23 (17.3%)	12 (16.9%)	1.1(0.5,2.5)	0.767
	Delta Sum > 0	133	71	16 (12.0%)	5 (7.0%)	1.9(0.5,7.2)	0.345
PF	Progression in any subregion	131	71	18(13.7%)	16(22.5%)	0.6(0.3,1.3)	0.176
	Delta Subregion > 0	128	71	17(13.3%)	15(21.1%)	0.6(0.3,1.3)	0.210
	Delta Sum > 0	128	71	10(7.8%)	10(14.1%)	0.5(0.2,1.9)	0.327
*MRI feature*	*Definition*	*T*	*P*	*T*	*P*	*RR (95% CI)*	*p-value*
Hoffa-synovitis/ Effusion-synovitis combined	Any worsening	136	71	13 (9.6%)	15 (21.1%)	0.5 (0.2,1.2)	0.115
Hoffa-synovitis	Any worsening	133	71	4 (3.0%)	5 (7.0%)	0.3 (0.1,1.8)	0.200
Effusion-synovitis	Any worsening	136	71	12 (8.8%)	11 (15.5%)	0.6 (0.2,2.0)	0.428

*T* treatment group, *P* placebo group, *TFJ* tibiofemoral joint, *RR* relative risk

^*a*^Note: We excluded knees with MRI score missing in any subregion at either baseline or follow-up visit when defining delta subregion and delta sum, but did not exclude these knees when defining progression in any subregion. So N used for progressoin in any subregion was equal to or larger than N used in the two delta approaches

A lower proportion of knees showed progression of Hoffa-synovitis and/or effusion synovitis (TG-C 9.6% vs. placebo 21.1%, *p* = 0.115), although the difference was not statistically significant.

Regarding change in BMLs and meniscal damage from baseline to 12 months, there was no significant difference using any of the three analytic approaches with similar rates of progression in both groups (TG-C vs. placebo: any BML progression 66.2% vs. 60.6%, *p* = 0.612; any meniscal damage progression, 31.6% vs. 32.4%, *p* = 0.993).

The Least Squared Mean Difference (95% CI) between the two groups in the osteophytes sum scores at the 12-month visit was 0.65 (−2.07 to 3.36), which was not statistically significant.

**Fig. 1:**
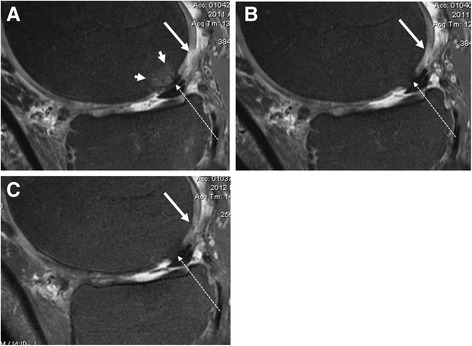
Sagittal intermediate-weighted fat suppressed MRI in treated patient at baseline (**a**), 3 months (**b**) and 6 months (**c**) follow up show improvement of cartilage focal defect of the posterior medial femoral condyle with almost perfect filling at 6 months (long arrows). Also note the grade 1 BML at the central weight-bearing medial femoral condyle disappears at 3 months (**a**, short arrows). There is a Hoffa-synovitis grade 1 and moderate size tibial osteophytes. There is a femoral intrachondral osteophyte that seems to be slightly increasing in size (dotted arrows)

**Fig. 2:**
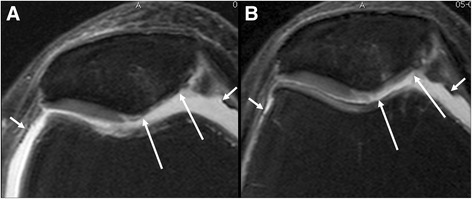
Axial intermediate-weighted fat-suppressed MRI in treated patient at baseline (**a**) and 12 months (**b**) follow up show improvement of cartilage focal defect and thickness of the medial patella (long arrows). Also note the decrease in volume of the joint effusion (small arrows)

**Fig. 3:**
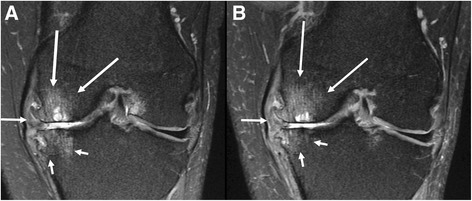
Coronal intermediate-weighted fat-suppressed MRI in treated patient at baseline (**a**) and 3 months (**b**) follow up show no improvement of cartilage damage in the medial tibiofemoral compartment (WORMS grade 6 at central medial femur; WORMS grade 5 at central medial tibia). There is a medial meniscal maceration/extrusion (arrow). There is a slight improvement of the subchondral bone marrow lesion/cysts at the central medial tibia (small arrows) and slight worsening of the subchondral bone marrow lesion/cysts at the central medial femur (long arrows). Note large medial osteophytes

Table 1 summarizes the details of the baseline to 12 months analyses for cartilage and inflammatory markers. Figures 1, 2 and 3 show examples of cases with positive effect of TG-C on MRI-depicted structural changes, as well as a case which did not show positive effect of TG-C.

## Discussion

In our phase II trial of allogeneic human chondrocytes expressing TGF-β1 (TG-C) vs. placebo in patients with moderate to advanced OA, a lower proportion of knees showed progression of cartilage damage on a knee level as well as less progression of MRI-based inflammatory markers for the treatment group compared to placebo. No preventive effect in regard to potential hypertrophic osteophyte formation was observed for the treatment group compared to placebo. Likewise, no significant differences between treatment and placebo were seen for change in BML and meniscal damage.

Previously, safety and tolerability of TG-C was demonstrated in a phase I human study [7]. More recently, positive effects of TG-C on knee function and pain in patients with moderate to advanced knee osteoarthritis were reported, showing some improvement in knee pain, function, and physical ability [3, 8, 9]. In these studies, however, MRI-based –structural assessment was not included.

MRI has become an essential research tool for knee OA studies thanks to its ability to non-invasively visualize the morphology of different knee joint tissues relevant to the disease process, and especially hyaline cartilage [10, 11]. Potential beneficial effects of TG-C on cartilage formation/growth for treatment of articular cartilage defects was first reported by Noh and colleagues in preclinical studies using mice, rabbits and goats [12]. In these animal studies, cartilage status after treatment was evaluated histologically at autopsy. Such an outcome measure is not appropriate in a phase II human study and therefore we deployed MRI which is a non-invasive imaging method.

Cho and colleagues reported a Korean phase IIa clinical trial in which MRI was used to evaluate effects of TG-C on structural changes including BMLs, cartilage damage, osteophytes, meniscal damage, effusion and periarticular inflammation 6-months and 12-months after TG-C injection [4]. In this study, effects of TG-C on MRI-based structural outcomes (assessed using the WORMS) were variable depending on TG-C dosage (low vs. high) and length of observation (6 months vs. 12 months). At 6 months, the low-dose cohort demonstrated worsening in mean MRI scores in one parameter (osteophytes), while the high-dose cohort demonstrated no worsening in mean scores. At 12 months, the low-dose cohort had worsening in the mean score in a subset of one parameter of unknown clinical significance, i.e. cartilage signal intensity, and the high-dose cohort demonstrated worsening in mean scores in two parameters (osteophytes and periarticular inflammation). This study was limited by a small number of subjects (*n* = 27), relatively short follow-up time and lack of a placebo group. The US phase II study described herein had more than double the sample size, had a placebo group, and deployed a more detailed outcome analyses.

Despite the fact that TGF-β1 is understood to be an anabolic agent, our study did not show any preventive effect of TG-C on progression of osteophyte formation in kneeOA. In fact, failing to reach statistical significance in all of our analyses may partly be due to sample size and low rates of progression overall. Also, since OA is a slowly progressing disease, a follow-up period of 12 months may have been too short to observe statistically significant effects of the TGF-β1 on structural progression of knee OA. Therefore, a future study with a larger number of subjects and a longer period of follow-up is necessary to confirm our preliminary observation regarding the effects of TGF-β1 on structural progression of knee OA. However, based on our results and thus far available literature evidence from animal and human studies, continued research efforts utilizing MRI-based structural outcomes to assess clinical efficacy of TG-C seem justifiable, especially focusing on cartilage regeneration.

## Conclusion

In conclusion, intraarticular treatment with TG-C may potentially show benefits on delayed progression of cartilage damage and MRI markers of inflammation in osteoarthritis with fewer patients in the treated group showing progression of these structural OA features. However, TG-C showed no preventive effect for progression of BMLs and meniscal damage, or hypertrophic osteophyte formation within 12 month follow-up.

## Abbreviations

BMI: Body mass index
BML: Bone marrow lesion
HIV: Human immunodeficiency virus
IKDC: International Knee Documentation Committee
MRI: Magnetic resonance imaging
OA: Osteoarthritis
TE: Time to echo
TG-C: Allogeneic human chondrocytes expressing TGF-β1
TGF: Transforming growth factor beta 1
TR: Time to repetition
VAS: Visual analogue scale
WORMS: Whole Organ Magnetic Resonance Imaging Score
